# Sixteen-year follow-up of childhood avalanche survivors

**DOI:** 10.3402/ejpt.v7.30995

**Published:** 2016-08-16

**Authors:** Edda Bjork Thordardottir, Unnur Anna Valdimarsdottir, Ingunn Hansdottir, Arna Hauksdóttir, Atle Dyregrov, Jillian C. Shipherd, Ask Elklit, Heidi Resnick, Berglind Gudmundsdottir

**Affiliations:** 1 Centre of Public Health Sciences, Faculty of Medicine, School of Health Sciences, University of Iceland, Reykjavik, Iceland; 2 Department of Epidemiology, Harvard School of Public Health, Boston, MA, USA; 3 Department of Medical Epidemiology and Biostatistics, Karolinska Institutet, Stockholm, Sweden; 4 Faculty of Psychology, School of Health Sciences, University of Iceland, Reykjavik, Iceland; 5 Center for Crisis Psychology, Bergen, Norway; 6 Department of Clinical Psychology, Faculty of Psychology, University of Bergen, Bergen, Norway; 7 National Center for PTSD, VA Boston Healthcare System, Boston, MA, USA; 8 Department of Psychiatry, Boston University School of Medicine, Boston, MA, USA; 9 Department of Psychology, University of Southern Denmark, Odense, Denmark; 10 Department of Psychology, University of Ulster, Londonderry, Northern Ireland; 11 Department of Psychiatry and Behavioral Sciences, Medical University of South Carolina, Charleston, SC, USA; 12 Mental Health Services, Landspitali – The National University Hospital of Iceland, Reykjavik, Iceland; 13 Faculty of Medicine, School of Health Sciences, University of Iceland, Reykjavik, Iceland

**Keywords:** Children, disaster, avalanche, posttraumatic stress disorder, long-term follow-up

## Abstract

**Background:**

Every year a substantial number of children are affected by natural disasters worldwide. However, data are scarce on long-term psychological impact of natural disasters on children's health. Identifying risk factors and outcomes associated with the long-term sequelae of posttraumatic stress disorder (PTSD) can provide a gateway to recovery as well as enhancement of preventive measures.

**Objective:**

Among childhood avalanche survivors, we aimed to investigate risk factors for PTSD symptoms and the relationship between socioeconomic status (SES) and PTSD symptoms in adulthood.

**Methods:**

Childhood survivors (aged 2–19 at the time of exposure) of two avalanches were identified through nationwide registers 16 years later. The Posttraumatic Diagnostic Scale was used to assess current PTSD symptoms. One-way ANOVA was used to explore PTSD symptoms by background and trauma-specific factors, as well as associations with current SES. Predictors of PTSD symptoms were examined by multivariable regression analysis.

**Results:**

Response rate was 66% (108/163). Results from univariate ANOVA analysis revealed that female sex was associated with PTSD symptoms (*F*=5.96, *p*<0.05). When adjusted for age and sex, PTSD symptoms were associated with lower education (*F*=7.62, *p*<0.001), poor financial status (*F*=12.21, *p*<0.001), and unemployment and/or disability (*F*=3.04, *p*<0.05). In a multivariable regression model, when adjusting for age and sex, lack of social support (*t*=4.22, *p*<0.001) and traumatic reactions of caregivers (*t*=2.49, *p*<0.05) in the aftermath of the disaster independently predicted PTSD 16 years post-trauma.

**Conclusions:**

Lingering PTSD symptoms after childhood exposure to a disaster may negatively influence socioeconomic development in adulthood. Strengthening children's support systems post-disaster may prevent the long-term sequelae of symptoms.

**Highlights of the article:**

- PTSD symptoms following avalanche exposure during childhood were associated with poorer socioeconomic status in adulthood.
- Lack of social support and traumatic reactions of caregivers in the aftermath of avalanches predicted PTSD symptoms among childhood survivors 16 years later.
- Findings underscore the importance of strengthening children's support systems in the aftermath of disasters.

Every year a substantial number of children are affected by natural disasters worldwide. Mental health research has increasingly focused on the detrimental consequences of disasters on the health of children and more recently has accentuated factors facilitating resilience and posttraumatic growth. Of mental health outcomes, posttraumatic stress disorder (PTSD) is likely the most prevalent post-disaster disorder affecting children (Hoven, Duarte, Turner, & Mandell, 2009). Longitudinal studies (>10 years) of childhood disaster survivors have shown the prevalence of PTSD to range from 2 to 29% (Boe, Holgersen, & Holen, 2011; Green et al., 1994; McFarlane & Van Hooff, 2009; Morgan, Scourfield, Williams, Jasper, & Lewis, 2003; Najarian, Sunday, Labruna, & Barry, 2011), indicating that factors other than the initial disaster exposure influence the progression of PTSD. In light of accumulating evidence of PTSD's adverse effects on multiple areas of children's lives, such as their social functioning and academic performance (Weems et al., 2013), identifying risk factors for PTSD has become an important area of research, providing a gateway to recovery as well as enhancement of preventive measures.

North's (2004) *disaster trauma theory* proposes a network of risk factors that contribute to post-disaster psychopathology, such as PTSD (Fig. 1). The model classifies risk factors into domains such as disaster agent characteristics (i.e., the subjective experience of the event and injury), individual characteristics (i.e., age and sex), and secondary sequelae (i.e., loss of social support).

**Fig. 1:**
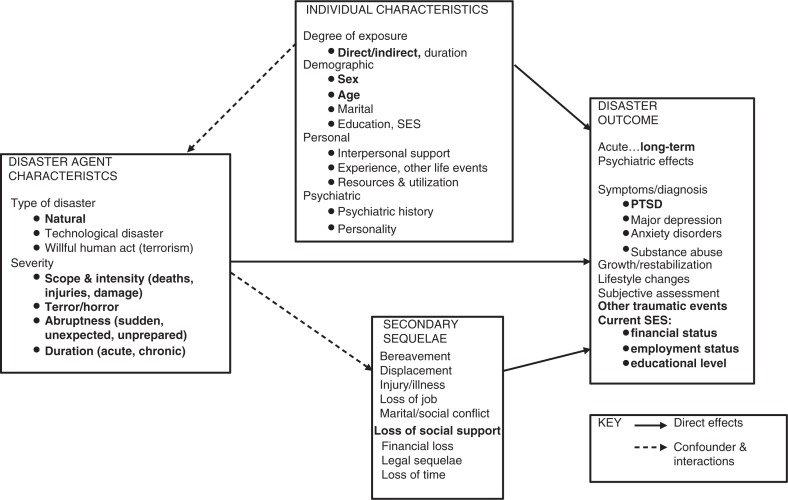
Modified version of North's (2004) disaster trauma theory. The domains “posttraumatic cognitive processing” and “community factors” are not presented in the figure. Current financial status, employment status, and education level are viewed as outcome variables as participants were children at the time of the disaster. Factors assessed in the current article are in bold.

An array of potential risk factors for PTSD has been identified among childhood disaster survivors. The literature has, however, been limited to a large extent to examining the first months and year post-disaster (Wang, Chan, & Ho, 2013). Of individual characteristics, previous studies indicate that females are at greater risk for PTSD than males, both in childhood and adolescence (Alisic et al., 2014). The literature on the relationship between age and PTSD among children has revealed mixed findings, as some studies indicate that the younger the children are, the greater the risk for developing PTSD (Brown, Mellman, Alfano, & Weems, 2011; Shannon, Lonigan, Finch, & Taylor, 1994), whereas others show older children experiencing greater symptom severity (Green et al., 1991). In line with this discrepancy, a meta-analysis of 96 child disaster studies found age at time of exposure to have a weak association with PTSD symptoms (Furr, Comer, Edmunds, & Kendall, 2010). In addition, greater number of lifetime traumatic events has been associated with increased PTSD risk, even when PTSD is assessed in relation to an index event. This relationship often appears in a dose-response fashion (Karam et al., 2014; Kilpatrick et al., 2013).

Disaster agent characteristics such as severity of the event and proximity to the disaster, loss of a loved one or friend (Furr et al., 2010), and injury (Adams et al., 2014) have been associated with PTSD symptoms in children. Furthermore, children's subjective experience of the disaster such as perceived life threat and distress at the time of the disaster has been found to yield medium-to-large associations with PTSD symptoms (Bodvarsdottir, Elklit, & Gudmundsdottir, 2006; Furr et al., 2010).

Children are a particularly vulnerable group in the post-disaster period and are likely to have difficulty coping with and recovering from the impact of disasters (Pfefferbaum, Noffsinger, Wind, & Allen, 2014). They are highly reliant on others for information and care and prior research has found low social support post-disaster to be a risk factor for PTSD among children (Trickey, Siddaway, Meiser-Stedman, Serpell, & Field, 2012). Caregivers serve as role models for coping, and parental psychopathology can have profound effects on children's recovery in the aftermath of disasters (Green et al., 1991).

In 1995, two small towns in the western part of Iceland were struck by avalanches without warning, destroying in total 48 houses and taking 34 lives (including 13 children). Previously, we found that 15% of childhood survivors at the time of the avalanches in the cohort under study reported PTSD symptoms (Posttraumatic Diagnostic Scale (PDS) score >14) within the past month at 16 years post-disaster (Thordardottir et al., 2016). The aim of the current study was to assess risk factors associated with PTSD symptoms in childhood survivors as well as the potential effects of PTSD symptoms on survivors’ socioeconomic status (SES) 16 years post-disaster. To our knowledge, this is the first study assessing risk factors for PTSD symptoms in childhood avalanche survivors. Adopting North's (2004) model as a framework, we hypothesized that female sex would be positively associated with PTSD symptoms. Because of the discrepancy in the literature, we did not expect to find an association between PTSD and a specific developmental period in childhood. In accordance with the disaster agent characteristics domain of North's model (2004), we predicted that being in town at the time of the avalanches and traumatic reactions (e.g., feeling intense fear or shock and helplessness), loss of a loved one, injury, and economic loss would be positively associated with PTSD symptoms. Finally, of secondary sequelae factors, we hypothesized that perceived lack of social support and traumatic reactions of caregivers would be positively associated with PTSD symptoms. Because the disaster occurred during participants’ childhood, other lifetime traumatic events and current SES were viewed as potential consequences of PTSD rather than risk factors for symptoms. Based on previous findings, we hypothesized that greater number of lifetime traumatic events and current SES, that is, low education, poor financial status, and unemployment and disability, would be positively associated with PTSD symptoms.

## Method

### Participants

Participants were 108 inhabitants of Sudavik and Flateyri in 1995 when the avalanches occurred, aged 2–19 at the time of the trauma. Residential records from 1995 with current contact information for previous residents of these towns were obtained from the Icelandic Bureau of Statistics. In 2011, we attempted to contact all residents who were 18 years or older in 2011 (2–19 years old in 1995), residing anywhere in Iceland with an active phone number or address (*N*=163). Response rate was 66% (108/163; 48% females, 52% males). More information about the data collection process is available in Thordardottir et al. (2015).

A previous study on the avalanche-affected populations found no difference between avalanche survivors and a non-exposed population with regard to SES (Thordardottir et al., 2015). When assessing childhood survivors specifically, we found no significant difference between the avalanche survivors and the non-exposed population with regard to current educational level (*χ*^2^ (2)=2.28, *p*=0.320), current personal finances (*χ*^2^ (2)=3.52, *p*=0.172), or current employment status (*χ*^2^ (2)=0.56, *p*=0.754). The characteristics of the unexposed population are reported in Thordardottir et al. (2015).

### Procedure

Approval for the study was granted by the National Bioethics Committee (VSNb2009080005/03.7). The Icelandic Data Protection Authority was notified of the study (s4608/2009/LSL/-). Participants received questionnaires via email or postal mail. Data were collected from January to June in 2011.

### Measures

We assessed sex and age, which was categorized into the following three groups: preschool (aged 2–5), grade school (aged 6–12), and adolescence (aged 13–19). Because the disaster occurred during participants’ childhood, current finances, current living situation, current employment status (classified as 1) working or student, 2) unemployed or on disability, and 3) parental leave or homemaker), and disability at any time after the avalanche were viewed as potential consequences of PTSD rather than risk factors for symptoms. Education was classified in line with the Icelandic school system, where grade school is compulsory for children 6 to 15 years old, immediately followed by a 4-year elective high school education. Education was divided into three groups: 1) university level, 2) high school or trade school, and 3) grade school or less education.

#### Disaster agent characteristics

Respondents were asked about losing a family member or close friend, sustaining injuries in the avalanche, suffering from economic loss (home or personal belongings partly or completely destroyed in the avalanche), their location when the avalanche struck (in or out of town), and their traumatic reactions during or in the aftermath of the trauma: 1) experienced their own life in danger, 2) experienced the life of a significant other in danger, 3) felt intense fear or shock, and 4) felt helplessness (reactions reflecting criterion A1 and A2 for PTSD in the fourth edition of the Diagnostic and Statistical Manual of Mental Disorders (American Psychiatric Association (APA), 2000)). Response options were yes/no. The relationship between respondents’ traumatic reactions and PTSD symptoms is shown in a univariate analysis, but in the multivariable analysis we created a summary index of respondents’ traumatic reactions (“own reaction”), which ranges on a scale from 0 to 4 (a sum of the number of participants’ own reactions).

#### Secondary sequelae

In line with the disaster model (North, 2004), we assessed perceived social support following the avalanche (“did you have someone to talk to in the aftermath of the avalanche?”) and who provided the support (response options: family, friend(s), local(s), a professional from the towns, an out-of-town professional). Respondents were asked whether their caregivers had shown the following traumatic reactions in the aftermath of the trauma: 1) emotional numbness, 2) feelings of intense fear or shock, 3) feeling of helplessness, 4) crying, and 5) outbursts of anger. Response options were yes/no. The relationship between respondents’ perception of their caregivers’ traumatic reactions and PTSD symptoms is tested in a univariate analysis, but in the multivariable analysis we created a summary index of caregiver traumatic reactions (“caregiver reaction”), which ranges on a scale from 0 to 5 (a sum of the number of caregiver reactions).

#### Posttraumatic stress symptoms

Lifetime non-avalanche-related traumatic events and posttraumatic stress symptoms reflecting DSM-IV (APA, 2000) were assessed using two of four sections of the PDS (Foa, Cashman, Jaycox, & Perry, 1997). The PDS is a 49-item self-report measure corresponding to all six criteria of the DSM-IV (APA, 2000). In the current study, we assessed the number of non-avalanche traumatic events (12 items) as well as PTSD symptoms (17 items). Reported history of traumatic events (excluding the avalanches in 1995) was assessed with the PDS and scored as 0–12, reflecting the number of traumatic events experienced or witnessed. With regard to PTSD symptom assessment, participants in this study responded to a modified version of the PDS tailored to the avalanches in 1995 (e.g., In the past month, have you been trying not to think about or have feelings associated with the avalanche in Flateyri/Sudavik?). Possible PTSD symptoms resulting from other traumatic events were not assessed. Questions on the PDS are scored from 0 (“not at all or only one time”) to 3 (“five or more times a week/ almost always”) for symptom frequency during the past month. Summing the ratings for all 17 symptoms produces total PTSD symptom severity scores (0–51). The scale has shown good concurrent validity and test–retest reliability for the 17 items reflecting PTSD symptoms (Foa, Riggs, Dancu, & Rothbaum, 1993). The Icelandic version has been shown to have good internal consistency for the 17 items reflecting PTSD symptoms (Ragnarsdottir & Gudmundsdottir, 2008). The coefficient α in the total cohort was 0.88.

### Statistical analysis

One-way ANOVA was used to test PTSD symptoms by background and trauma-specific factors, as well as associations with current SES. Multivariable regression analysis with simultaneous entry of variables was used to examine predictors of PTSD symptoms. Covariates included in the multivariable regression model were all factors statistically significant in prior age- and sex-adjusted models. Adjusting for other lifetime traumatic events in the final model did not affect results (data not shown). The statistical program IBM SPSS Statistics version 20.0 was used to conduct analyses.

## Results

Females were significantly more likely to experience PTSD symptoms than males. No group difference was found within other individual factors, that is, developmental stage at time of trauma and current living situation (Table 1). When adjusted for age and sex, PTSD symptoms were associated with low educational level, poor financial status, unemployment and/or disability, and greater number of lifetime traumatic events (Table 1).

**Table 1:** *F*-values from ANOVAs that examined the effects of current demographic factors on current PTSD symptoms in childhood avalanche survivors

	PDS score			
	
Individual characteristics	*N* (%)	Mean (SD)	*F*-value	Adjusted *F*-value^a^
Sex				
Male	56 (52)	5.0 (6.2)	5.96*	−
Female	52 (48)	8.3 (7.5)		
Age in years at time of trauma				
2–5	30 (28)	5.6 (6.5)	0.92	−
6–12	31 (29)	6.5 (7.3)		
13–19	47 (44)	7.3 (7.2)		
Current educational level				
University	22 (20)	4.3 (5.2)	8.74**	7.62***
High school or trade school	48 (44)	5.4 (6.4)		
Grade school or less educ.	38 (35)	9.4 (7.9)		
Current personal finances				
Very good or good	34 (32)	2.9 (4.5)	23.39***	12.21***
Moderate ends meet	54 (51)	6.9 (7.0)		
Poor or very poor	19 (18)	11.8 (7.3)		
Current living situation				
Married or in relationship	73 (68)	6.7 (7.0)	0.02	2.28
Single, divorced	35 (32)	6.5 (7.3)		
Current employment status				
Working or student	88 (82)	5.9 (6.8)	3.79	3.04*
Unemployed/on disability	13 (12)	11.1 (7.9)		
Parental leave/homemaker	7 (7)	8.3 (6.0)		
Nr. lifetime traumatic events^b^				
0	31 (33)	4.9 (5.4)	4.19*	3.58*
1–2	39 (42)	7.3 (6.8)		
3 or more	23 (25)	8.7 (8.9)		

a Adjusted for age and sex.

b Avalanches in 1995 excluded.

* *p*<0.05

** *p*<0.01

*** *p*<0.001.

PTSD symptoms were associated with the following disaster agent characteristics: loss of a family member or close friend in relation to the avalanche and being in town at the time of the trauma. Of participants’ own reaction to the disaster, experiencing their life in danger, and feelings of helplessness in the aftermath of the trauma were associated with PTSD symptoms. PTSD symptoms were not significantly related to other disaster agent characteristics, that is, experiencing the life of significant others in danger and feeling of intense fear or shock, having experienced injuries or economic loss (Table 2).

**Table 2:** *F*-values from ANOVAs that examined the association of risk factors and current PTSD symptoms in childhood avalanche survivors

	PDS score			
	
	*N* (%)	Mean (SD)	*F*-value	Adjusted *F*-value^a^
Disaster agent characteristics				
Lost family member or close friend				
No	24 (23)	4.6 (5.3)	2.61	2.93*
Yes	80 (77)	7.3 (7.4)		
Sustained injury				
No	74 (91)	7.1 (7.0)	0.22	2.01
Yes	7 (9)	8.4 (8.8)		
Economic loss^b^				
No	54 (57)	6.7 (7.2)	0.01	1.75
Yes	41 (43)	6.8 (7.2)		
In town at time of avalanche				
No	23 (22)	4.4 (6.4)	2.79	3.57*
Yes	81 (78)	7.2 (7.1)		
Own reaction				
Own life in danger				
No	78 (75)	5.1 (5.8)	16.79***	6.69***
Yes	26 (25)	11.3 (8.5)		
Life of significant other in danger				
No	27 (26)	5.3 (5.1)	1.32	2.54
Yes	77 (74)	7.2 (7.6)		
Feelings of intense fear or shock				
No	34 (34)	5.8 (7.2)	0.79	2.10
Yes	66 (66)	7.2 (7.0)		
Feelings of helplessness				
No	40 (39)	5.0 (6.3)	4.16*	2.94*
Yes	62 (61)	7.9 (7.4)		
Secondary sequelae factors				
Social support in aftermath of avalanche				
No	24 (26)	11.3 (8.3)	14.18***	7.32***
Yes	67 (74)	5.3 (6.1)		
Traumatic reaction from caregivers				
Emotional numbness				
No	27 (30)	3.3 (6.34)	11.61**	5.59**
Yes	63 (70)	8.6 (6.95)		
Feelings of intense fear or shock				
No	25 (28)	3.7 (6.07)	7.39**	5.32**
Yes	63 (72)	8.1 (7.01)		
Feeling of helplessness				
No	24 (27)	4.8 (5.71)	2.98	3.39*
Yes	64 (73)	7.6 (7.32)		
Crying				
No	17 (19)	6.5 (7.92)	0.12	1.99
Yes	72 (81)	7.2 (7.07)		
Outbursts of anger				
No	48 (54)	5.7 (6.20)	2.66	3.06*
Yes	40 (46)	8.2 (7.74)		

a Adjusted for age and sex.

b Home, business or personal belongings partly or completely destroyed in the avalanche.

* *p*<0.05

** *p*<0.01

*** *p*<0.001.

Testing of the model's (North, 2004) secondary sequelae factors revealed that when adjusted for age and sex, PTSD symptoms were associated with lack of social support in the aftermath of the avalanche. Of those who received social support, 93% received support from their parents, 91% from their friends, 3% from a professional in the disaster area, and 18% from an out-of-town professional. In addition, of caregivers’ traumatic reactions, as reported by childhood survivors, showing emotional numbness, feelings of intense fear or shock, feelings of helplessness, and outbursts of anger were associated with respondents’ current PTSD symptoms. No association was observed between having caregivers who cried in the aftermath of the trauma and respondents’ PTSD symptoms (Table 2).

When disaster-related factors were examined in a multivariable regression analysis (adjusted for age and sex), reported lack of social support in the aftermath of the disaster and traumatic reactions of caregivers independently predicted PTSD symptoms among childhood survivors 16 years post-trauma (Table 3).

**Table 3:** Multivariable linear regression between disaster-related risk factors and current PTSD symptoms in childhood avalanche survivors

	B	*t*	95% CI for B	*p*
Young age	−0.13	−0.86	−0.43; 0.17	0.39
Female sex	2.90	2.09	0.14; 5.66	0.04
Lost family member or close friend	1.32	0.76	−2.15; 4.79	0.45
In town when avalanche hit	−0.39	−0.23	−3.77; 2.98	0.82
Lack of social support	6.51	4.22	3.44; 9.58	0.00
Caregiver traumatic reaction	1.22	2.49	0.24; 2.20	0.02
Own traumatic reaction	0.68	1.01	−0.66; 2.02	0.31

B, unstandardized regression coefficient; CI, confidence interval.

The relationship between caregiver traumatic reactions and PTSD symptoms remains statistically significant (*F* (1, 198)=17.48, *p*<0.001) when participants who were 2–5 years old at the time of the trauma are excluded from analysis.

## Discussion

In our 16-year follow-up of childhood avalanche survivors, we found lack of social support and traumatic reactions of caregivers (as perceived by respondents) in the aftermath of the disaster to uniquely predict current PTSD symptoms. Furthermore, we found that PTSD symptoms were associated with lower education, poor financial status, and unemployment and disability in adulthood. Our study addresses gaps in the literature, as it is the first population-based cohort study assessing risk factors for PTSD symptoms among childhood survivors of avalanche exposure.

Our results are consistent with North's model (2004) and previous research which has found a supportive environment post-trauma to be a strong indicator of recovery (Masten & Narayan, 2012; Pfefferbaum, Jacobs, Houston, & Griffin, 2015; Spell et al., 2008). These findings are consistent with a prospective study of young hurricane survivors (La Greca, Silverman, Lai, & Jaccard, 2010) where high levels of social support from family and friends had a buffering effect on PTSD in childhood disaster survivors at a 9- and 21-month time point post-disaster.

Our findings indicate that caregivers who reportedly showed traumatic reactions (e.g., numbness, intense fear, and shock) in the aftermath of the disaster impacted respondents’ PTSD symptoms in adulthood. However, it is also important to note that caregiver crying in the aftermath of the avalanche (a normative response to emotions) was not associated with PTSD in childhood survivors. Caregivers are usually children's main form of support in the post-disaster period, and previous research has found that their adaptive functioning impacts their children's stability (Trickey et al., 2012). It is noteworthy that in our multivariable analysis, caregivers’ traumatic reactions, for example, numbness, intense fear, and shock (as perceived by the childhood survivors), were a greater predictor of PTSD symptoms, than childhood survivors own traumatic reactions. In line with our results, previous studies have found parents’ trauma-related distress to negatively impact children's well-being post-trauma (Lambert, Holzer, & Hasbun, 2014).

Furthermore, consistent with our results, Dyb, Jensen, and Nygaard (2011) concluded that the explained variance of PTSD scores in children is more related to family level variance factors (such as parental exposure, parental immediate reactions, and posttraumatic stress reactions) than individual variance factors (e.g., the child's age and sex). Caregivers’ reactions have previously been found to influence children's coping behaviors (Spell et al., 2008). Witnessing caregivers crying, as opposed to showing emotional numbness, might prompt children to express their feelings rather than internalize them, possibly explaining why we found no relationship between caregivers response of crying in the aftermath of the disaster and respondents’ PTSD symptoms.

It is possible that the association between caregivers’ traumatic reactions and childhood survivor PTSD in our study is mediated by caregivers’ PTSD, as reported by previous findings (Foy, Madvig, Pynoos, & Camilleri, 1996). It is also possible that the association between low SES status and childhood survivors PTSD symptoms is confounded by other factors such as parental SES status. However, to secure anonymity of participants, we did not obtain information about family ties within the avalanche cohort, preventing us from exploring the association between caregivers’ and childhood survivors’ PTSD symptoms.

Our study provides evidence that PTSD symptoms are inversely associated with educational level, consistent with previous research, which has found PTSD to be associated with school absence and poor school performance among disaster-affected children (Broberg, Dyregrov, & Lilled, 2005; Weems et al., 2013). It is possible that traumatic stress affects brain development, with evidence suggesting that traumatic stress early in life impacts critical areas of learning, such as memory processing and executive function (Brewin, 2011; Carrion & Wong, 2012; Scrimin, Moscardino, Capello, & Axia, 2009). In line with these findings, Dyregrov, Dyregrov, Endsjø, and Idsoe (2015) found that teachers often perceive deterioration in school performance among traumatized children, particularly difficulty in concentration. It is therefore particularly important that post-disaster interventions focus on equipping schools, a cornerstone in children's development, with means to support those traumatized, both academically and socially. Our results further indicate that PTSD symptoms adversely affect financial status and the ability to work in adulthood, as previously evidenced in long-term studies in other childhood trauma populations (Zielinski, 2009).

In addition, we found that additional lifetime trauma was associated with increased PTSD symptoms among respondents. These results are in line with previous studies, which have found PTSD symptoms attributed to many events to be associated with elevated hyperarousal symptoms and longer duration of symptoms (Karam et al., 2014).

The main strength of this study is that it is a complete, population-based follow-up of a childhood population exposed to a natural disaster. Through the Icelandic nationwide register, we were able to identify all survivors residing in the country 16 years post-disaster. The questionnaire was developed following qualitative interviews with the avalanche survivors and the previous literature. In addition, the use of anonymous self-administered questionnaires decreased risk of interview-related bias. However, despite the relatively high response rate for a disaster survivor study, the health status of those who chose not to participate remains unknown.

Because of the retrospective nature of our study, we cannot be certain about the temporal sequence of the factors under study. Therefore, the associations observed in our study lend important hints subject to replication in prospective studies. However, it is likely that the onset of PTSD symptoms was in the first months post-disaster, as previous research indicates that late onset of PTSD is uncommon (Smid, Mooren, van der Mast, Gersons, & Kleber, 2009; Yule et al., 2000). Retrospective studies are subject to recall bias, and because of the young age of some respondents at the time of the trauma (28% were ≤5 years old) and the time elapsed since the disaster, events may not be adequately recollected. In addition, the severity of the event may introduce a risk of memory distortion. Some studies have indicated that the retention of events accompanied by extreme stress is more vulnerable to error than events accompanied by moderate stress (Fivush, McDermott Sales, Goldberg, Bahrick, & Parker, 2004; Morgan & Southwick, 2014), although further research in this area is needed (Price & Connolly, 2008).

Furthermore, reporting of perceived social support and caregivers’ traumatic reactions might not solely reflect acute reactions but rather responses and support received in the long-term. As many individuals have difficulty recalling events occurring early in life, this might particularly apply to the youngest participants in our study. It is also possible that poor social support post-disaster reflects respondents’ social environment pre-disaster. Similarly, caregivers’ traumatic reactions might be an indicator of psychopathological problems among caregivers pre-disaster. Nonetheless, whether parental distress or poor support systems were present before or after the disaster, our results highlight the importance of bolstering children's support system and supporting caregivers in helping their children in the post-disaster period.

Disasters are social events that need to be viewed in the context of communities. Our results suggest that it is of critical importance for disaster interventions to focus on assisting families. Supporting primary caregivers in their role as well as enhancing the availability of social support in the community for children (such as in the school environment) is crucial to their recovery in the long term (Broberg et al., 2005). The early intervention program “Child and Family Stress Intervention” has, for instance, been found to prevent the development of chronic PTSD in trauma-exposed children. The program aims at increasing communication about feelings and symptoms between the trauma-exposed child and their caregivers, as well as teaching behavioral skills to both the caregiver and child (Berkowitz, Stover, & Marans, 2011). Furthermore, it appears to be just as important for interventions to focus on alleviating caregivers own distress symptoms as well as the child's symptoms, as the former may have a large impact on children's recovery process. Lastly, having evidence-based PTSD therapy available to children who go on to develop the disorder post-disaster is essential to the alleviation of symptoms, as evidenced by previous research (Goenjian et al., 2005).

## Conclusion

This study indicates that PTSD symptoms following a disaster during childhood may have a negative effect on SES in adulthood. In addition, results highlight the need for strengthening children's support systems in the aftermath of disasters to prevent the long-term sequelae of symptoms. Offering evidence-based and affordable treatment to this particularly vulnerable group is an important public health matter that needs to be addressed, funded, and available in the long term.

## References

[CIT0001] Adams Z.W, Sumner J.A, Danielson C.K, McCauley J.L, Resnick H.S, Gros K, Ruggiero K.J (2014). Prevalence and predictors of PTSD and depression among adolescent victims of the Spring 2011 tornado outbreak. Journal of Child Psychology and Psychiatry.

[CIT0002] Alisic E, Zalta A.K, van Wesel F, Larsen S.E, Hafstad G.S, Hassanpour K, Smid G.E (2014). Rates of posttraumatic stress disorder in trauma-exposed children and adolescents: Meta-analysis. British Journal of Psychiatry.

[CIT0003] American Psychiatric Association (2000). Diagnostic and Statistical Manual of Mental Disorders.

[CIT0004] Berkowitz S.J, Stover C.S, Marans S.R (2011). The child and family traumatic stress intervention: Secondary prevention for youth at risk of developing PTSD. Journal of Child Psychology and Psychiatry and Allied Disciplines.

[CIT0005] Bodvarsdottir I, Elklit A, Gudmundsdottir D.B (2006). Posttraumatic stress reactions in children after two large earthquakes in Iceland. Nordic Psychology.

[CIT0006] Boe H.J, Holgersen K.H, Holen A (2011). Mental health outcomes and predictors of chronic disorders after the North Sea oil rig disaster: 27-year longitudinal follow-up study. Journal of Nervous and Mental Disease.

[CIT0007] Brewin C.R (2011). The nature and significance of memory disturbance in posttraumatic stress disorder. Annual Review of Clinical Psychology.

[CIT0008] Broberg A.G, Dyregrov A, Lilled L (2005). The Goteborg discotheque fire: Posttraumatic stress, and school adjustment as reported by the primary victims 18 months later. Journal of Child Psychology and Psychiatry.

[CIT0009] Brown T.H, Mellman T.A, Alfano C.A, Weems C.F (2011). Sleep fears, sleep disturbance, and PTSD symptoms in minority youth exposed to Hurricane Katrina. Journal of Traumatic Stress.

[CIT0010] Carrion V.G, Wong S.S (2012). Can traumatic stress alter the brain? Understanding the implications of early trauma on brain development and learning. Journal of Adolescent Health.

[CIT0011] Dyb G, Jensen T.K, Nygaard E (2011). Children's and parents’ posttraumatic stress reactions after the 2004 tsunami. Clinical Child Psychology and Psychiatry.

[CIT0012] Dyregrov A, Dyregrov K, Endsjø M, Idsoe T (2015). Teachers’ perception of bereaved children's academic performance. Advances in School Mental Health Promotion.

[CIT0013] Fivush R, McDermott Sales J, Goldberg A, Bahrick L, Parker J (2004). Weathering the storm: Children's long-term recall of hurricane Andrew. Memory (Hove, England).

[CIT0014] Foa E.B, Cashman L, Jaycox L, Perry K (1997). The validation of a self-report measure of posttraumatic stress disorder: The posttraumatic diagnostic scale. Psychological Assessment.

[CIT0015] Foa E.B, Riggs D.S, Dancu C.V, Rothbaum B.O (1993). Reliability and validity of a brief instrument for assessing posttraumatic stress disorder. Journal of Traumatic Stress.

[CIT0016] Foy D.W, Madvig B.T, Pynoos R.S, Camilleri A.J (1996). Etiologic factors in the development of posttraumatic stress disorder in children and adolescents. Journal of School Psychology.

[CIT0017] Furr J.M, Comer J.S, Edmunds J.M, Kendall P.C (2010). Disasters and youth: A meta-analytic examination of posttraumatic stress. Journal of Consulting and Clinical Psychology.

[CIT0018] Goenjian A.K, Walling D, Steinberg A.M, Karayan I, Najarian L.M, Pynoos R (2005). A prospective study of posttraumatic stress and depressive reactions among treated and untreated adolescents 5 years after a catastrophic disaster. American Journal of Psychiatry.

[CIT0019] Green B.L, Grace M.C, Vary M.G, Kramer T.L, Gleser G.C, Leonard A.C (1994). Children of disaster in the second decade: A 17-year follow-up of Buffalo Creek survivors. Journal of the American Academy of Child and Adolescent Psychiatry.

[CIT0020] Green B.L, Korol M, Grace M.C, Vary M.G, Leonard A.C, Gleser G.C, Smitson-Cohen S (1991). Children and disaster: Age, gender, and parental effects on PTSD symptoms. Journal of the American Academy of Child and Adolescent Psychiatry.

[CIT0021] Hoven C.W, Duarte C.S, Turner J.B, Mandell D.J, Neria Y, Galea S, Norris F.H (2009). Child mental health in the aftermath of disasters: A review of PTSD studies. Mental Health and Disasters.

[CIT0022] Karam E.G, Friedman M.J, Hill E.D, Kessler R.C, McLaughlin K.A, Petukhova M, Koenen K.C (2014). Cumulative traumas and risk thresholds: 12-month PTSD in the World Mental Health (WMH) surveys. Depression and Anxiety.

[CIT0023] Kilpatrick D.G, Resnick H.S, Milanak M.E, Miller M.W, Keyes K.M, Friedman M.J (2013). National estimates of exposure to traumatic events and PTSD prevalence using DSM-IV and DSM-5 Criteria. Journal of Traumatic Stress.

[CIT0024] La Greca A.M, Silverman W.K, Lai B, Jaccard J (2010). Hurricane-related exposure experiences and stressors, other life events, and social support: Concurrent and prospective impact on children's persistent posttraumatic stress symptoms. Journal of Consulting and Clinical Psychology.

[CIT0025] Lambert J.E, Holzer J, Hasbun A (2014). Association between parents’ PTSD severity and children's psychological distress: A meta-analysis. Journal of Traumatic Stress.

[CIT0026] Masten A.S, Narayan A.J (2012). Child development in the context of disaster, war, and terrorism: Pathways of risk and resilience. Annual Review of Psychology.

[CIT0027] McFarlane A.C, Van Hooff M (2009). Impact of childhood exposure to a natural disaster on adult mental health: 20-year longitudinal follow-up study. British Journal of Psychiatry.

[CIT0028] Morgan C.A, Southwick S (2014). Perspective: I believe what I remember, but it may not be true. Neurobiology of Learning and Memory.

[CIT0029] Morgan L, Scourfield J, Williams D, Jasper A, Lewis G (2003). The Aberfan disaster: 33-year follow-up of survivors. British Journal of Psychiatry.

[CIT0030] Najarian L.M, Sunday S, Labruna V, Barry I (2011). Twenty-year follow-up of adults traumatized during childhood in Armenia. Journal of Affective Disorders.

[CIT0031] North C.S, Gorman M. Jack (2004). Psychiatric effects of disasters and terrorism: Empirical basis from study of the Oklahoma City bombing. Fear and anxiety: The benefits of translational research.

[CIT0032] Pfefferbaum B, Jacobs A.K, Houston J.B, Griffin N (2015). Children's disaster reactions: The influence of family and social factors. Current Psychiatry Reports.

[CIT0033] Pfefferbaum B, Noffsinger M.A, Wind L.H, Allen J.R (2014). Children's coping in the context of disasters and terrorism. Journal of Loss and Trauma.

[CIT0034] Price H.L, Connolly D.A (2008). Children's recall of emotionally arousing, repeated events: A review and call for further investigation. International Journal of Law and Psychiatry.

[CIT0035] Ragnarsdottir M, Gudmundsdottir M.O (2008). Prevalence of traumatic events and posttraumatic stress disorder among university students.

[CIT0036] Scrimin S, Moscardino U, Capello F, Axia G (2009). Attention and memory in school-age children surviving the terrorist attack in Beslan, Russia. Journal of Clinical Child and Adolescent Psychology.

[CIT0037] Shannon M.P, Lonigan C.J, Finch A.J, Taylor C.M (1994). Children exposed to disaster: I. Epidemiology of posttraumatic symptoms and symptom profiles. Journal of the American Academy of Child and Adolescent Psychiatry.

[CIT0038] Smid G.E, Mooren T.T.M, van der Mast R.C, Gersons B.P.R, Kleber R.J (2009). Delayed posttraumatic stress disorder: Systematic review, meta-analysis, and meta-regression analysis of prospective studies. The Journal of Clinical Psychiatry.

[CIT0039] Spell A.W, Kelley M.L, Wang J, Self-Brown S, Davidson K.L, Pellegrin A, Baumeister A (2008). The moderating effects of maternal psychopathology on children's adjustment post-hurricane Katrina. Journal of Clinical Child and Adolescent Psychology.

[CIT0046] Thordardottir E.B, Hansdottir I, Shipherd J.C, Valdimarsdottir U.A, Resnick H, Elklit A, Gudmundsdottir R, Gudmundsdottir B (2016). Risk factors for posttraumatic stress symptoms among avalanche survivors: A 16 year follow-up. Journal of Nervous and Mental Disease.

[CIT0040] Thordardottir E.B, Valdimarsdottir U.V, Hansdottir I, Resnick H, Shipherd J.C, Gudmundsdottir B (2015). Posttraumatic stress and other health consequences of catastrophic avalanches: A 16-year follow-up of survivors. Journal of Anxiety Disorders.

[CIT0041] Trickey D, Siddaway A.P, Meiser-Stedman R, Serpell L, Field A.P (2012). A meta-analysis of risk factors for posttraumatic stress disorder in children and adolescents. Clinical Psychology Review.

[CIT0042] Wang C.W, Chan C.L, Ho R.T (2013). Prevalence and trajectory of psychopathology among child and adolescent survivors of disasters: A systematic review of epidemiological studies across 1987–2011. Social Psychiatry and Psychiatric Epidemiology.

[CIT0043] Weems C.F, Scott B.G, Taylor L.K, Cannon M.F, Romano D.M, Perry A.M (2013). A theoretical model of continuity in anxiety and links to academic achievement in disaster-exposed school children. Development and Psychopathology.

[CIT0044] Yule W, Bolton D, Udwin O, Boyle S, O'Ryan D, Nurrish J (2000). The Long-term psychological effects of a disaster experienced in adolescence: I: The incidence and course of PTSD. Journal of Child Psychology and Psychiatry.

[CIT0045] Zielinski D.S (2009). Child maltreatment and adult socioeconomic well-being. Child Abuse and Neglect.

